# Supplementary material for: Systematic Generation of Patient-Derived Tumor Models in Pancreatic Cancer
Source: Cells. 2019 Feb 10;8(2):142. doi: 10.3390/cells8020142 (PMC6406729; doi:10.3390/cells8020142)
Supplement: Supplementary file 1 [file cells-08-00142-s001.pdf]

Supplementary Materials

Supplementary Methods

Methods S1: Step-by-step protocol for the establishment of pancreatic cancer primary cultures.

Collection and handling of pancreatic cancer patients' primary tumor tissues

1. Upon surgical resection, collect tumor tissue in 1xPBS at 4°C and immediately transport it into the lab hood for further processing.

❖ ***Critical step: Immediately proceed with tumor processing to reduce the loss of viable tumor cells.***

2. Cut off a representative piece of tumor and store it in 10% formalin for pathological assessment under sterile conditions.

3. Cut tumors into pieces <1g for xenotransplantation into the flank of immune deficient NOD.Cg-*Prkdc*^{scid} Il2rg^{tm1Wjl}/SzJ (NSG) mice.

In vivo xenotransplantation of tumor pieces

1. Wash tumor piece for xenotransplantation three times with 25 mL 1xPBS/PS (0.1 mg/mL penicillin/streptomycin in 1xPBS).
2. Anesthetize the mouse according to ethics approval.
3. Weigh the mouse and subcutaneously inject 10 µl Carprofen solution (0.4 µg/µl) per gram of bodyweight as painkiller to the right mid-abdominal site.
4. Lay the anesthetized mouse on the warming pad to maintain body temperature. Shave its body hair at the left flank with a medical scalpel and disinfect the shaved area with alcohol pads.
5. Make a perpendicular incision of approximately 1.0 cm in the disinfected area and create a bag-like space by bracing scissors or tongs below the skin. Gently insert the cut tumor piece into the bag.
6. Close the incision by pressing both wound sites together. Then clamp the wound with 2–3 metal clips and disinfect this area with a propanol and polyvidone-iodine containing solution.
7. Uniquely mark each transplanted mouse.
8. Closely examine transplanted mice according to ethics approval. Remove the metal clips after 10 days.

Purification of pancreatic cancer tissue

This protocol describes the purification of primary patient tumor tissue as well as tumor tissue in vivo expanded in the immunodeficient NSG mice:

1. Mince the tumor tissue under sterile conditions into approximately 1 mm^3 pieces and resuspend them in 20 mL 1xPBS/PS and centrifuge at 900 rpm for 5 min (4 °C). Wash 3 times with 20 mL 1x PBS/PS three times.

❖ ***Critical step: Tumor tissues should be minced as small as possible to increase surface for subsequent digestion.***

2. Resuspend the tumor pieces with 20 mL digestion medium and incubate for 30 minutes up to 150 minutes at 37 °C under constant rotation. Digestion medium: Medium199 with 5% 40 mg/mL Collagenase IV in PBS and 0.6% 25 mM $CaCl_2$ in H_2O.

❖ ***Critical step: Tumor incubation should be checked every 30 minutes to avoid total digestion of tumor. The digestion should be terminated before most tumor pieces are fully dissolved.***

3. Next, thoroughly pipet the suspension up and down and filter through a 100 μm cell strainer. Centrifuge the flow-through at 1000 rpm for 10 minutes at 4 °C. Using a cell scraper, collect all tumor pieces from the filter into a 50 mL Falcon tube. Resuspend the tumor pieces in a 12.5 mL serum-free cancer stem cell new medium (CSCN) and transfer them to an adherent T75 cell culture flask to establish the matching in vitro primary cell culture (details shown in next section).

❖ ***Critical step: 12 mL serum-free CSCN medium should be changed within the next 24 hours. Closely check if any sign of contamination is visible. If so, the cell culture should be discarded.***

4. Discard the supernatant after the centrifugation and resuspend the cell pellets in 10 mL 1xPBS/PS. Then repeat the filtering step twice with a Falcon® 40 μm cell strainer.
5. Calculate the number of purified tumor cells and subcutaneously transplant into immunodeficient mice (0.2 $x10^6$ to 7 $x10^6$ cells), or freeze in liquid nitrogen for long term storage.

Serial xenotransplantation of purified tumor cells

1. 1:1 mix the purified tumor cells with Matrigel to a maximal volume of 50 μL.
   - ❖ ***Critical step: All the processes should be executed fast as Matrigel solidifies quickly at room temperature. Use a 1 mL syringe with blunt (1.2 mm x 40 mm) needle to transfer the Matrigel drop into a 500 μL tube containing purified cell pellets. The volume ratio of Matrigel and cell pellets in medium is 1:1. Gently mix the cell suspension with the blunt needle.***
2. Transfer the cell/Matrigel mixture into an injection syringe with 0.4 mm fine needle (27 G) for further xenotransplantation. Store the syringe at 4 °C during transportation.

3. Anesthetize the mouse according to the ethical approval standards.
4. Weigh the mouse and subcutaneously inject 10 µL Carprofen solution (0.4 µg/µL) per gram of bodyweight to the right mid-abdominal site.

   Lay the anesthetized mouse on the warming pad to maintain body temperature. Use an alcohol pad to disinfect the right flank area.
5. Lift the disinfected area by two fingers and inject the cell-Matrigel mixture into the space between the skin and muscle layer. A small knob below the skin at the injection site indicates that the purified tumor cells were successfully transplanted.

❖ ***Critical step: Remove the needle and wait until the knob of solidified Matrigel is visible under the skin and then put the mouse back in the cage. Check its physical activity before terminating the xenotransplantation.***

Establishment and daily handling of pancreatic cancer primary cultures

1. After tumor digestion, transfer the undigested tumor pieces in 12 mL serum-free CSCN medium into an adherent T75 cell culture flask. CSCN medium is routinely used for the establishment and daily culturing of primary cultures.

   ❖ ***Critical step: Freshly prepare the sterile CSCN medium for generation and daily cultivation of patient-derived cell cultures.***
2. Add 10 ng/mL recombinant human basic fibroblast growth factor (rhFGF), 20 ng/mL rhFGF10 and 20 ng/mL rhNodal to the culture medium.
3. Within the next days to weeks, depending on the individual patient cells, closely monitor the primary cell culture for the growth of adherent colonies. Unattached tumor pieces can optionally be removed once a sufficient amount of primary tumor cells grow out. Primary tumor cells form adherent colonies while maintaining tight cell-to-cell contact. Change the CSCN medium at least twice per week or when a change in color is observed. Add 10 ng/mL rhFGF, 20 ng/mL rhFGF10 and 20 ng/mL rhNodal twice per week or when CSCN medium is changed.
4. Cell passaging

   Cells should be passaged when the growing viable cells cover >70% of the flask surface.

   a) Remove the CSCN medium and wash cells with 10 mL 1XPBS.

   b) Add accutase into the cell flask (e.g. 5 mL in adherent T75 flask) and incubate for 10 to 30 minutes at 37 °C.

❖ ***Critical step: Accutase is a relatively mild digestive enzyme. Check if all cells are detached every 5–10 minutes after it is added into cell flask.***

   c) Add 10 mL 1XPBS to the cell suspension when all cells are detached. Centrifuge the cell suspension at 1200 rpm for 5 minutes.

   d) Remove the supernatant and resuspend the cells with fresh CSCN medium.

Replate 1/6 to 1/3 the cells into a new flask and add 10 ng/mL basic rhFGF, 20 ng/mL rhFGF10 and 20 ng/mL rhNodal. Freeze the rest of cells.

5. Cell freezing

   a) Detach the cells and prepare the cell pellets as described above.

   b) Resuspend $1x10^5$ to $1x10^7$ cells in 700 µL RPMI medium and transfer into one cell-freezing tube. Add the same amount of freshly prepared freezing medium and gentle mix it with cell suspension.

   c) Put all tubes into an isopropanol-filled cell freezing container with 1 °C/min cooling rate and leave it at –80 °C for 24 hours. Then transfer the frozen cells into liquid nitrogen for long-term storage.

6. Cell thawing

   a) Take the cell freezing tube from liquid nitrogen and directly warm it up between your hand palms until liquid is near completely thawn or in a 37 °C water bath.

   b) Add one drop of the thawing medium to the cell freezing tube as the cells are almost completely thawed. Then, transfer all the cell solution into an empty 50 mL tube within 20 seconds.

   c) Add 2 mL thawing medium to the tube within 20 seconds. Repeat the same procedure with 4 mL and 8 mL thawing medium at each step.

   ❖ ***Critical step: To prevent any physical harm to the cells, add the thawing medium at low speed (last for 20 seconds at each step) and let it flow down at one side of the tube wall. Avoid directly dropping the medium on the cells.***

   d) Centrifuge the cell solution at 1200 rpm, 4 °C for 5 minutes. Discard the supernatant and resuspend the cell pellets with 10 mL 1xPBS.

   e) Centrifuge the cell solution at 1200 rpm, 4 °C for 5 minutes. Discard the supernatant and resuspend the cell pellets with 12 mL CSCN medium with cytokines.

   f) Plate cells as described above.

7. Composition of medium

   a) The Cancer Stem Cell New (CSCN) medium

   500 mL advanced DMEM-F12 added with 3.0 g D-Glucose, 2% B27-supplement (1x), 1% L-glutamine (2 mM), 0.5% HEPES buffer (5 mM) and 6.0 mg Heparin sodium salt.

   b) Digestion medium

   95% Medium 199 with 5% Collagenase IV (2 mg/mL) and 0.6% $CaCl_2$ (3 mM)

   c) Cell freezing medium

   55% RPMI medium with 30% FBS and 15% DMSO

   d) Cell thawing medium

50% CSCN medium and 50% FBS

Supplementary Figures

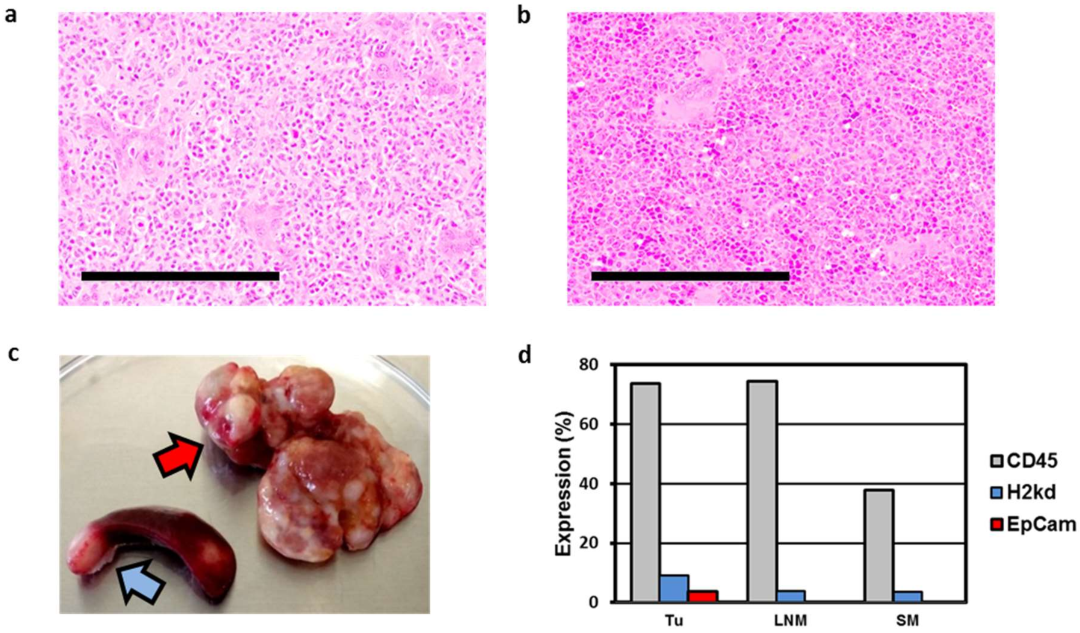

Figure 1. Xenograft and metastasis dominated by outgrow of CD45+ cells. (**a**) H.E. staining of liver and (**b**) spleen metastasis generated by CD45+ cells. (**c**) CD45(+) cells are responsible for extended spleen (blue arrow) and liver metastasis (red arrow). (**d**) Proportion of epithelial (EpCam+), hematopoietic (CD45+) and murine (H2kd+) cells of purified B-lymphoma contaminated expansion xenograft including primary tumor (TU), lymph node metastasis (LNM) and spleen metastasis (SM). Despite domination by CD45+ cells, the primary transplantation site still shows low EpCam expression, whereas metastasis is completely negative for EpCam. Scale bars: 200 µm. (a, b): PC 32; (c): PC 41; (d): PC 29.

Supplementary Tables

Table S1. Phenotypic characterization of primary PC cultures with stromal morphology.

Marker	Patient F1	Patient F2
**Flow Cytometry**		
CD133	0.0%	0.0%
CD44	99.9%	97.7%
EpCam	0.6%	0.1%
CD24	1.1%	0.0%
**Indirect Immunofluorescence**		
KRT7	+	n.d.
Vimentin	+	+
αSMA	+	+
THY1	+	+
Nestin	+/-	+/-
Desmin	-	-
Klf4	+	+

Representative flow cytometry analysis of stroma cultures from patients F1 and F2 reveals high CD44, low EpCam and CD24, and no detectable CD133 expression. Cells are positive for mesenchymal stroma markers vimentin, α-smooth-muscle actin (α-SMA) and THY1. Patient F1 culture co-expresses the pancreatic duct marker KRT7. Cultures contain single nestin+ cells but are mainly negative for desmin. KLF4 is expressed throughout examined cultures. Cultures were assigned + when positive cells were detected in at least two independent visual fields of the same sample, +/– when only single positive cells were identified and – if no positive stained cells were detectable; n.d. = not determined.

Table S2. Patient characteristics.

Patient number	Age (yr)	Gender	Histology	UICC-Stage	Weight of patient sample
PC1	62	Male	Ductal adenocarcinoma	IIB	0.10 g
PC2	70	Female	Ductal adenocarcinoma	IIB	1.10 g
PC3	74	Male	Adenosquamous carcinoma	IIB	N/A
PC4	53	Female	Peritoneal adenocarcinoma	IV	N/A
PC5	69	Female	Ductal adenocarcinoma	IIA	0.30 g
PC6	68	Male	Ductal adenocarcinoma	IIB	1.0 g
PC7	76	Female	Ductal adenocarcinoma	IIB	1.70 g
PC8	72	Male	Ductal adenocarcinoma	IV	1.05 g
PC9	77	Female	Ductal adenocarcinoma	IIB	N/A
PC10	62	Male	Ductal adenocarcinoma	IIB	0.90 g
PC11	72	Male	Ductal adenocarcinoma	IIB	0.30 g
PC12	62	Male	Ductal adenocarcinoma with oncocytic type IPMN	IIA	0.76 g
PC13	66	Male	Adenosquamous carcinoma	IIA	0.52 g
PC14	82	Female	Ductal adenocarcinoma	IIB	0.84 g
PC15	86	Male	Adenosquamous carcinoma	IIB	0.25 g
PC16	62	Male	Ductal adenocarcinoma	IV	0.68 g
PC17	69	Male	Ductal adenocarcinoma	III	0.25 g
PC18	52	Female	Ductal adenocarcinoma	IIB	0.95 g
PC19	67	Female	Ductal adenocarcinoma	IIB	0.26 g
PC20	66	Female	Ductal adenocarcinoma	IV	0.40 g
PC21	72	Male	Ductal adenocarcinoma	IIB	0.40 g
PC22	74	Male	Ductal adenocarcinoma	IIB	0.40 g
PC23	78	Female	Mucinous ductal adenocarcinoma	IIB	0.40 g
PC24	83	Male	Ductal adenocarcinoma	IIB	0.40 g
PC25	62	Male	Ductal adenocarcinoma	IIB	0.56 g
PC26	65	Female	Ductal adenocarcinoma	IIB	0.17 g
PC27	83	Male	Ductal adenocarcinoma	IIB	0.65 g
PC28	63	Female	Ductal adenocarcinoma	IIB	0.43 g
PC29	61	Male	Distal bile duct adenocarcinoma	IIB	0.67 g
PC30	60	Male	Ductal adenocarcinoma	IIB	1.1 g
PC31	60	Male	Ductal adenocarcinoma	IV	0.85 g
PC32	70	Female	Ductal adenocarcinoma	IIB	0.40 g
PC33	48	Male	Ductal adenocarcinoma	IIA	0.87 g
PC34	65	Male	Ductal adenocarcinoma	IIA	2.50 g
PC35	49	Female	Ductal adenocarcinoma	IIB	1.50 g
PC36	67	Female	Ductal adenocarcinoma	IIB	1.40 g
PC37	75	Female	Ductal adenocarcinoma	IIB	0.25 g
PC38	88	Male	Signet ring adenocarcinoma	IIB	2.60 g
PC39	61	Female	Ductal adenocarcinoma	IIB	1.10 g
PC40	58	Female	Ductal adenocarcinoma	IIB	0.60 g
PC41	60	Male	Ductal adenocarcinoma	IIB	0.99 g
PC42	78	Male	Mucinous (colloidal) adenocarcinoma	IIA	2.10 g
PC43	66	Male	Ductal adenocarcinoma	IIB	1.00 g
PC44	56	Male	Ductal adenocarcinoma	IIA	0.50 g

PC45	52	Male	Ductal adenocarcinoma	III	0.80 g
PC46	70	Female	Ductal adenocarcinoma	IIB	1.55 g
PC47	66	Female	Ductal adenocarcinoma	IIB	0.20 g
PC48	63	Male	Ductal adenocarcinoma	IIB	1.23 g
PC49	61	Female	Ductal adenocarcinoma	IV	0.30 g
PC50	58	Male	Ductal adenocarcinoma	IV	0.88 g
PC51	77	Female	Main-duct IPMN (Benign)	Not applicable	0.95 g
PC52	48	Male	Ductal adenocarcinoma	IIB	0.89 g
PC53	65	Male	Ductal adenocarcinoma	IIB	0.84g
PC54	62	Female	Branch-duct IPMN (Benign)	Not applicable	0.31 g
F1	66	Male	Ductal adenocarcinoma	IIA	N/A
F2	77	Female	Ductal adenocarcinoma	IIA	N/A

IPMN: Intraductal papillary mucinous neoplasm.

Table S3. Marker expression of CD45(+) xenografts.

Patient number	EpCam	H2kd	CD45
**PC29**	3.7%	9.0%	73.6%
**PC32**	0.1%	17,3%	76.6%
**PC41**	0%	24.7%	64.9%

EpCam: human epithelial cell marker; H2kd: murine cell marker; CD45: human leukocyte marker.

Table S4. Marker expression of established primary PC cultures.

Patient number	EpCam	H2kd	CD45	Matching cell culture	
				Successfully established	Murine cell contamination
**PC7**	99.7%	0.1%	0.0%	+	
**PC8**	98.0%	0.2%	0.2%	+	
**PC11**	91.2%	10.8%	0.0%	+	
**PC13**	97.7%	2.0%	0.0%	+	
**PC17**	84.0%	0.0%	0.0%	+	
**PC20**	99.9%	0.1%	0.1%	+	
**PC21**	99.9%	0.1%	0.1%	+	
**PC22**	92.9%	4.6%	0.1%	+	
**PC25**	98.5%	0.1%	0.1%	+	
**PC28**	97.3%	4.1%	1.4%	+	
**PC30**	99.7%	0.0%	0.0%	+	
**PC35**	98.9%	0.0%	0.1%	+	
**PC15**	0.2%	97.9%	0.1%		+
**PC19**	0.4%	99.8%	0.1%		+
**PC23**	0.3%	65.9%	38.0%		+
**PC26**	32.2%	70.5%	0.5%		+
**PC27**	0.5%	96.0%	1.8%		+
**PC31**	0.1%	98.8%	0.1%		+
